# Adenosine Amine Congener as a Cochlear Rescue Agent

**DOI:** 10.1155/2014/841489

**Published:** 2014-08-26

**Authors:** Srdjan M. Vlajkovic, Hao Chang, Song Yee Paek, Howard H.-T. Chi, Sreevalsan Sreebhavan, Ravindra S. Telang, Malcolm Tingle, Gary D. Housley, Peter R. Thorne

**Affiliations:** ^1^Department of Physiology, Faculty of Medical and Health Sciences, The University of Auckland, Private Bag 92019, Auckland 1142, New Zealand; ^2^Centre for Brain Research, Faculty of Medical and Health Sciences, The University of Auckland, Private Bag 92019, Auckland 1142, New Zealand; ^3^Section of Audiology, Faculty of Medical and Health Sciences, The University of Auckland, Private Bag 92019, Auckland 1142, New Zealand; ^4^Auckland Cancer Society Research Centre, Faculty of Medical and Health Sciences, The University of Auckland, Private Bag 92019, Auckland 1142, New Zealand; ^5^Department of Pharmacology, Faculty of Medical and Health Sciences, The University of Auckland, Private Bag 92019, Auckland 1142, New Zealand; ^6^Translational Neuroscience Facility and Department of Physiology, School of Medical Sciences, UNSW Australia, Sydney, NSW 2052, Australia

## Abstract

We have previously shown that adenosine amine congener (ADAC), a selective A_1_ adenosine receptor agonist, can ameliorate noise- and cisplatin-induced cochlear injury. Here we demonstrate the dose-dependent rescue effects of ADAC on noise-induced cochlear injury in a rat model and establish the time window for treatment. *Methods*. ADAC (25–300 *μ*g/kg) was administered intraperitoneally to Wistar rats (8–10 weeks old) at intervals (6–72 hours) after exposure to traumatic noise (8–16 kHz, 110 dB sound pressure level, 2 hours). Hearing sensitivity was assessed using auditory brainstem responses (ABR) before and 12 days after noise exposure. Pharmacokinetic studies investigated ADAC concentrations in plasma after systemic (intravenous) administration. *Results*. ADAC was most effective in the first 24 hours after noise exposure at doses >50 *μ*g/kg, providing up to 21 dB protection (averaged across 8–28 kHz). Pharmacokinetic studies demonstrated a short (5 min) half-life of ADAC in plasma after intravenous administration without detection of degradation products. *Conclusion*. Our data show that ADAC mitigates noise-induced hearing loss in a dose- and time-dependent manner, but further studies are required to establish its translation as a clinical otological treatment.

## 1. Introduction

Hearing loss is one of the greatest causes of disability (WHO), affecting up to 1 in 6 of the population. It is estimated that approximately 20% of the burden is generated from excessive noise exposure in occupational and leisure settings [1]. Hearing loss comes at a great economic cost (estimated at 1.6–3.2% GDP in Australia; Access Economics Report, 2006) and reduces the quality of life of the affected individuals. On a personal level, hearing loss results in considerable communication difficulties, social isolation, and depression and appears to be associated with early cognitive decline [2]. The damage from noise exposure is cumulative over time and exacerbates the effects of aging on hearing loss.

Noise-induced hearing loss (NIHL) is particularly common in the military, in industrial settings (construction workers, mining, forestry, and aircraft industry), and in the music industry. The proportion of nonwork related NIHL is also considered to be on the rise contributing significantly to the overall impact of NIHL. Hearing conservation programs are generally ineffective [3], as there are many instances of unprotected exposure to excessive noise, particularly in the military and heavy industry. Cumulative NIHL associated with recreational activities and loud music from personal listening devices is also contributing to the growth in hearing disability. Sensory hair cells in the cochlea damaged by noise do not regenerate, so their loss is permanent. Prosthetic rehabilitation via hearing aids and cochlear implants is the only current treatment for hearing loss from inner ear injury and these have significant limitations. It is therefore important to develop therapies that can prevent or repair injury to the delicate inner ear structures rather than relying upon medical devices that boost residual hearing functionality. We and others have identified adenosine receptors as one of the most promising targets for the treatment of NIHL.

Adenosine is a cytoprotective substance released from tissues in response to stress. Acting on adenosine receptors (AR), adenosine augments antioxidant defences, increases oxygen supply, improves blood flow, inhibits the release of neurotransmitters, stabilises cells by stimulating K^+^ channels and inhibiting Ca^2+^ channels, triggers anti-inflammatory responses, and promotes antiapoptotic pathways [4–6]. Neuroprotective actions of adenosine receptors in CNS disorders such as stroke, epilepsy, migraine, neurodegenerative, and neuropsychiatric disorders have been well documented [7–9]. Four distinct adenosine receptor subtypes have been characterised and designated as A_1_, A_2A_, A_2B_, and A_3_ [10]. Selective AR agonists are now being developed as cardioprotective and neuroprotective (A_1_ and A_3_), anti-inflammatory (A_2A_ and A_3_), and antinociceptive (A_1_) agents, whilst AR antagonists show therapeutic potential as neuroprotective (A_2A_) and antiglaucoma (A_3_) agents [10]. Some of AR agonists either are FDA approved or are currently being investigated in clinical trials [11].

All four adenosine receptors are expressed in the mammalian cochlea and they are differentially distributed in cochlear tissues [12]. Immunohistochemistry demonstrates that the sensory hair cells, supporting Deiters' cells and spiral ganglion neurons, express multiple adenosine receptors [12]. The sources of extracellular adenosine in cochlear fluids include active transport from the intracellular compartment by nucleoside transporters, adenosine release from damaged cells, and extracellular ATP hydrolysis [13, 14]. Adenosine activates adenosine receptors on target cells in a paracrine or autocrine fashion, whilst the clearance of adenosine from the extracellular space is provided by nucleoside transporters [14].

Previous studies have shown that prophylactic treatment with adenosine receptor agonists mitigates hearing loss from noise [15, 16] and the anticancer drug cisplatin [17]. We have shown that the local or systemic administration of selective A_1_ adenosine receptor (A_1_R) agonists, such as 2-chloro-*N* ^6^-cyclopentyladenosine (CCPA) and adenosine amine congener (ADAC), can ameliorate cochlear injury and hearing loss following noise exposure [18, 19]. The important aspect of this finding is that the drugs were administered after the cessation of noise exposure, suggesting that A_1_R agonists could be useful for the treatment of acute noise-induced cochlear injury within a therapeutic window, and not only as prophylactics. In contrast to other drugs acting on A_1_R, ADAC lacks cardiovascular side effects at the dose used to treat NIHL in these experiments [19–21], which suggests its suitability for systemic administration. The lack of systemic side effects within the therapeutic dose range for NIHL is due to a modified chemical structure, and its increased ability to cross the blood-brain barrier [22, 23] likely reflects permeability across the blood/perilymph partition within the cochlea. We previously showed that a five-day treatment of daily ADAC injections, starting six hours after exposure to noise, rescued up to 25 dB of otherwise permanent hearing loss [19]. For reference, 10 dB rescue in hearing thresholds is considered clinically significant [24]. The improvement of hearing thresholds was supported by increased survival of sensory hair cells and reduced expression of oxidative stress markers in the cochlea. We have also shown that ADAC ameliorates cisplatin-induced cochlear injury and hearing loss [25].

In the present study, using a rat model, we demonstrate the time window for ADAC otoprotective treatment after noise exposure and the optimal doses for systemic administration. Pharmacokinetic studies demonstrate changes in ADAC concentrations in plasma after systemic administration. These data provide a background for drug development studies which aim to establish the suitability of ADAC as a treatment for acute NIHL.

## 2. Materials and Methods

### 2.1. Animals

The studies were performed on male Wistar rats (8–10 weeks old) sourced from the animal facility at the University of Auckland. All procedures in this study were approved by the University of Auckland Animal Ethics Committee and conformed to international guidelines for the ethical use of animals. After completion of manipulations, animals were euthanised using sodium pentobarbital (100 mg/kg, i.p.) and cochlear tissues collected for histology.

### 2.2. Noise Exposure

Rats were exposed to 8–16 kHz octave band noise for 2 hours at 110 dB SPL to induce permanent hearing loss in untreated animals. Noise exposures were carried out in a custom-built acoustic chamber (Shelburg Acoustics, Sydney, Australia) with internal speakers and external controls (sound generator and frequency selector). The sound intensity inside the chamber was measured using a calibrated Bruel & Kjaer 2232 sound level meter to ensure minimal deviations of sound intensity. Control animals were housed in the animal facility at ambient sound conditions (45–55 dB SPL, 0.5–20 kHz).

### 2.3. Auditory Brainstem Responses (ABR)

ABR thresholds in response to 8–28 kHz tone pips were measured in a sound attenuating chamber (Shelburg Acoustics, Sydney, Australia) before and 12 days after noise exposure. Rats were anaesthetised with a mixture of ketamine (90 mg/kg) and xylazine (10 mg/kg) intraperitoneally and then placed onto a heating pad, to maintain body temperature at 37°C. ABRs were obtained by placing fine platinum electrodes subdermally at the mastoid region of the ear of interest (active electrode), scalp vertex (reference), and mastoid region of the opposite ear (ground electrode). The acoustic stimuli were supplied via a TD48 Beyer dynamic transducer connected to a 10 cm plastic tube that was placed into the external auditory canal of the left ear. A Tucker-Davis Technology (TDT) auditory physiology workstation System 3 (Alachua, FL, USA), equipped with a computer-based digital signal processing package and software (BioSig, Alachua), was used to produce the acoustic stimuli and record the ABR responses. Tone pips (5 ms, 0.5 ms rise-fall time) were presented at frequencies between 8 and 28 kHz at varying intensity levels. The threshold of the ABR complex (waves I–V) were determined by progressively attenuating the sound intensity in 5 dB steps until the wave I–V complex of the averaged ABR waveforms (1024 repeats with stimulus polarity alternated) was no longer distinguishable from noise floor in recorded traces. The ABR threshold was defined as the lowest intensity (to the nearest 5 dB) at which a response could be visually detected above the noise floor. Repeat waveforms were analysed at each frequency to determine the consistency of the responses and to identify the recurring peaks.

### 2.4. ADAC Treatment

ADAC (Sigma-Aldrich) was dissolved in 1 M HCl and then in 0.1 M phosphate buffered saline (PBS; pH 7.4) to prepare a 100 *μ*g/mL stock solution. The stock solution was then aliquoted and stored at −20°C for later use. Light-protected ADAC aliquots were thawed at 37°C for 30 min before administration. In study one, the following single ADAC dosages were used to optimize the rescue dose: 25, 50, 100, 200, and 300 *μ*g/kg/day. An equal volume of vehicle solution was given to control animals. ADAC or control vehicle solution was administered intraperitoneally (i.p.) for five consecutive days at 24 h intervals, beginning six hours after the cessation of noise exposure. In study two, one of the higher ADAC doses (200 *μ*g/kg) was used to determine the time window for treatment after noise exposure. ADAC treatment (five daily injections at 24 h intervals) commenced at 12, 24, 48, or 72 hours after noise exposure.

### 2.5. Measurement of ADAC Concentrations in Rat Plasma after Intravenous Administration

Male Wistar rats (8 weeks old) were anaesthetised with ketamine (90 mg/kg i.p.) plus xylazine (10 mg/kg i.p.) and the femoral vein was surgically exposed. ADAC (400 *μ*g/kg) was injected (1 mL/min) through the femoral vein, and blood samples (0.5 mL) were taken from the heart. Up to 1.5 mL of blood was taken from each animal, and the first sample was drawn 1 min after drug delivery. Subsequent extraction and purification procedures were modified from Stocchi et al. [26]. Briefly, after deproteination with 0.1 M KOH, plasma was extracted from the blood using an Amicon Ultra-4 50 K centrifugal filtering device (Merck Millipore, Tullagreen, Carrigtwohill, IRL). The filtrate was mixed with 50 *μ*L of 1 M KH_2_PO_4_ and subjected to HPLC analysis using an Agilent 1100 series instrument (Agilent Technologies, Santa Clara, California, USA). Reverse phase HPLC separation of ADAC was achieved on a Phenomenex Gemini 3 *μ*m C18 column (150 × 3.00 mm, 110 Å) protected with a Phenomenex Gemini C18 guard column (4 × 2.0 mm i.d.). The mobile phase used for the separation of ADAC from the matrix peaks consisted of two eluents: 45 mM ammonium formate in water, pH 7 (aqueous mobile phase A), and 100% acetonitrile (organic mobile phase B). The chromatographic conditions are shown in (Table 1).

Peaks were recorded with a diode-array detector at 254 nm detection wavelength and identified by comparison of retention times (RT) with the standard. Integration of peak areas was performed using Chemstation software version B.01.03 (Agilent).

### 2.6. Data Analysis

Results are presented as the mean ± SEM and the α level was set at *P* = 0.05. Each set of posttreatment threshold shifts compared between the control group and ADAC-treated groups using one-way ANOVA and post hoc Holm-Sidak multiple pairwise comparison test. Pharmacokinetic (PK) data were analysed by PKSolver [27].

## 3. Results

### 3.1. Dose-Response Study

**Figure 1:**
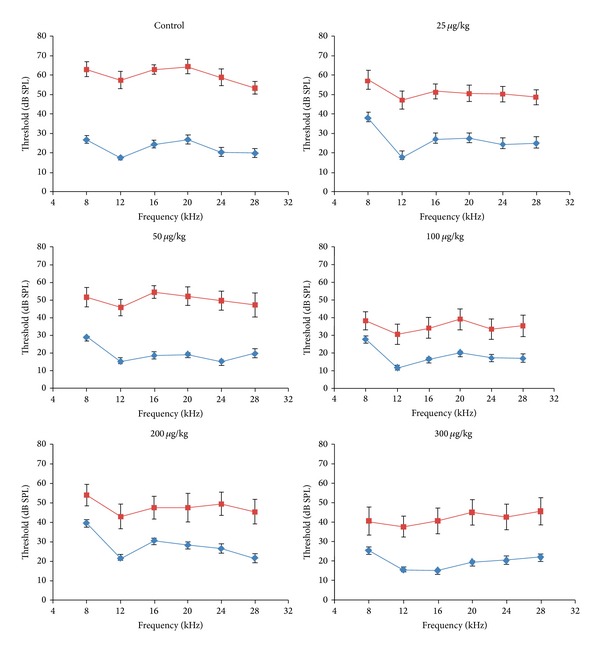
ADAC dose-response study in Wistar rats: the effect on auditory brainstem responses (ABR) before (blue line) and 12 days after (red line) traumatic noise exposure (8–16 kHz, 110 dB SPL, 2 hours). ADAC (25–300 *μ*g/kg) was administered intraperitoneally for five consecutive days at 24 h intervals, commencing six hours after noise exposure. In the control group, injections of the vehicle solution were administered at the same intervals as ADAC. ABR were measured in response to tone pips (8–28 kHz). Data are expressed as mean ± SEM (*n* = 8–10).

**Figure 2:**
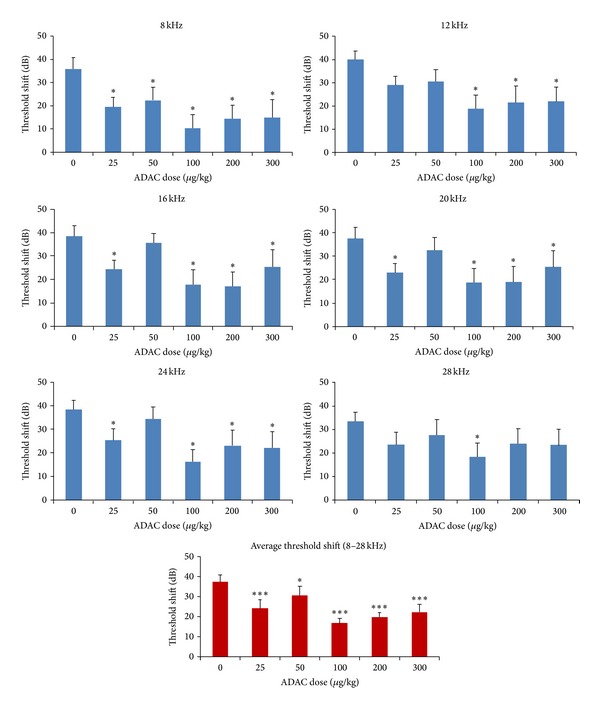
ADAC dose-response study: the effect on noise-induced ABR threshold shifts (defined as the difference between ABR thresholds before and after noise exposure at a single frequency). Threshold shifts averaged across all frequencies (8–28 kHz) are also shown. ADAC (25–300 *μ*g/kg i.p.) was administered for five consecutive days at 24 h intervals, commencing six hours after noise exposure. In the control group (ADAC dose “0”), injections of the vehicle solution were administered at the same intervals as ADAC. Data are expressed as mean ± SEM (*n* = 8–10). *P < 0.05; ***P < 0.001 versus control, one-way ANOVA with pairwise comparison.

ADAC (25–300 *μ*g/kg) was administered to noise-exposed Wistar rats for five consecutive days, commencing six hours after noise exposure (8–16 kHz, 110 dB SPL for 2 hours). Auditory thresholds were assessed using auditory brainstem responses (ABR) before and 12 days after noise exposure.  Figure 1 shows the baseline and final ABR thresholds in ADAC- and vehicle-treated (control) rats. Baseline ABR thresholds were comparable in all groups. All ADAC-treated groups showed a broad reduction of final thresholds across the 8–28 kHz frequency range compared with the hearing loss in the control group. ABR threshold shifts for each frequency are shown in Figure 2. In the control noise-exposed and nontreated group, the average threshold shift across the frequencies was 37 dB, with the largest shift of 40 dB at 12 kHz. In this study, all ADAC doses significantly (*P* < 0.05) reduced the extent of the threshold shift at some or all frequencies. The most effective ADAC doses (100 *μ*g/kg and 200 *μ*g/kg) reduced average noise-induced threshold shift across the frequencies by 21 dB and 18 dB, respectively (*P* < 0.001; ANOVA). These two ADAC doses significantly (*P* < 0.05) reduced threshold shifts at 8–24 kHz frequencies, but thresholds at the highest frequency (28 kHz) improved only with a 100 *μ*g/kg ADAC dose. The 300 *μ*g/kg and 25 *μ*g/kg doses were also effective, reducing average threshold shift by 15 dB and 13 dB, respectively (Figure 2). The 50 *μ*g/kg ADAC doses were the least effective, reducing average threshold shifts by 7 dB (*P* < 0.05). The effect of this dose was significantly (*P* < 0.01) lower compared to ADAC doses over 100 *μ*g/kg.

### 3.2. ADAC Efficacy Study

**Figure 3:**
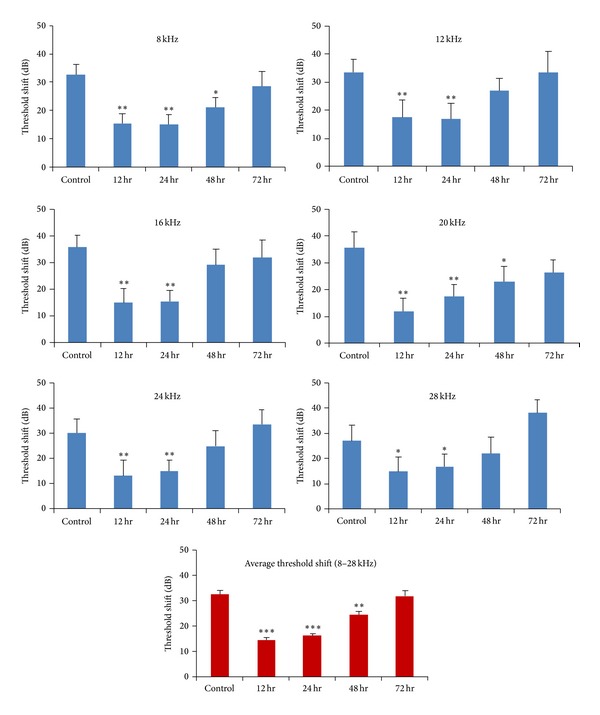
ADAC efficacy study. ADAC (200 *μ*g/kg i.p.) was administered as 5 daily injections commencing 12, 24, 48, or 72 hours after traumatic noise exposure, and the outcomes were measured using auditory brainstem responses (ABR) before and 12 days after noise exposure. In the control group, injections of the vehicle solution were administered at the same intervals as ADAC starting 12 hours after noise exposure. Data are expressed as ABR threshold shifts at different frequencies (mean ± SEM; *n* = 8–10) and threshold shifts averaged across all frequencies (8–28 kHz). *P < 0.05; **P < 0.01; ***P < 0.001 versus control, one-way ANOVA with pairwise comparison.

The optimal time to commencement of treatment was investigated using 5 daily ADAC injections (200 *μ*g/kg) commencing 12, 24, 48, or 72 hours after traumatic noise exposure (Figure 3). The outcomes were measured using ABR before and 12 days after noise exposure. ADAC treatment significantly (*P* < 0.001) reduced ABR threshold shifts in the treatment groups that commenced 12 and 24 hours postnoise exposure by 18 dB and 16 dB, respectively, averaged across the measured frequencies. The treatment regime that commenced 48 hours after noise exposure produced an average protection of 8 dB, which was also statistically significant (*P* < 0.01). At 72 hours delay before commencing ADAC treatment, the threshold shifts were similar to nontreated animals. This study suggests that ADAC treatment is most effective in the first 24 hours after noise exposure.

### 3.3. Pharmacokinetic Properties of ADAC after Systemic Administration

**Figure 4:**
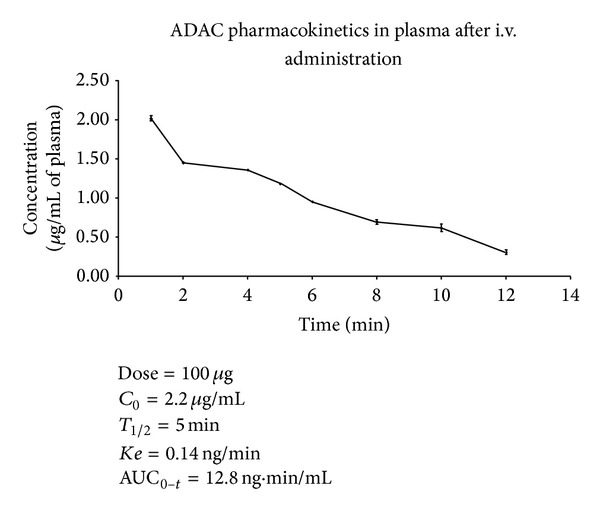
Pharmacokinetic properties of ADAC in plasma after administration through the femoral vein (400 *μ*g/kg, 1 mL/min). Data are expressed as mean ± SEM (*n* = 4 per time point). Pharmacokinetic properties were calculated using an Excel plugin PKSolver [24]. *C*_0_, extrapolated maximum concentration; *T*_1/2_, half-life; *Ke*, elimination constant; AUC_0–*t*_, the integral area under the curve, measuring the overall amount of ADAC in the compartment.

ADAC concentrations in plasma were determined using HPLC analysis. As expected, ADAC concentration was highest shortly after i.v. administration, followed by a rapid distribution and elimination phase (Figure 4), but without detection of degradation products. The graph shows concentration changes of ADAC in rat plasma for the time period of 12 minutes; after that, ADAC concentrations dropped below the limit of UV-Vis detection (~0.1 *μ*g/mL). The pharmacokinetic properties of ADAC in plasma were analysed by PKSolver, an add-in program for pharmacokinetic data analysis in Microsoft Excel [27]. Figure 4 shows that ADAC has a short (5 min) half-life (*T*_1/2_) in plasma and a fast elimination rate (*Ke*). The cochlear tissue was also analysed for ADAC in these experiments using UV-Vis detection. ADAC was detected at 5 min after injection in 4 out of 6 cochleae obtained from 3 animals but at 15 min postinjection ADAC was no longer detectable (data not shown). More sensitive assay is required to accurately assess ADAC concentrations in cochlear perilymph.

## 4. Discussion

This study shows that ADAC mitigates noise-induced hearing loss in a dose- and time-dependent manner. The ADAC was effective across the broad dose range, from 25 *μ*g/kg–300 *μ*g/kg i.p., reducing noise-induced threshold shifts by clinically significant levels [24] across the tested frequency range. At some frequencies, threshold improvement was up to 25 dB (Figure 2). ADAC was most effective at rescuing NIHL when treatment commenced within 24 hours after noise exposure. After 48 hours, ADAC improved ABR thresholds by more than 10 dB at two frequencies, 8 kHz and 20 kHz, suggesting that even delayed treatment could be useful. Pharmacokinetic studies demonstrated a short half-life of ADAC in plasma after intravenous administration without detection of degradation products. This rapid clearance suggests broad uptake into tissue compartments.

The dose-response curve for individual ADAC doses was nonlinear (nonmonotonic). The doses over 100 *μ*g/kg were the most effective in reducing threshold shifts, followed by 25 and 50 *μ*g/kg. Nonmonotonic dose response curves are often U-shaped or inverted U-shape (biphasic) but can also show complex multiphasic shape [28]. Similar nonmonotonic dose-response curves were observed in gerbils, in the study investigating the neuroprotective effect of ADAC in experimentally induced cerebral ischaemia [21]. In that study, acute prophylactic administration of ADAC at doses ranging from 25 to 200 *μ*g/kg was effective in preserving neurons, apart from the 50 *μ*g/kg dose, which failed to improve neuronal survival. In the same study, chronic treatment with ADAC was effective at lower doses: 10–100 *μ*g/kg in reducing mortality, and 25–100 *μ*g/kg in neuronal preservation. Another study showed that an acute treatment with ADAC (100 *μ*g/kg) is strongly neuroprotective in a model of Huntington's disease [29], whilst chronic administration at the same dose was ineffective. Together with our results, these studies provide a good indication of the neuro- and otoprotective dose range for ADAC, with the caveats such as different dosing schedules and disease models, and possible species-related differences in tissue distribution and affinity of A_1_R.

The present study also demonstrates pharmacokinetic properties of ADAC in rat plasma. The ADAC concentration curve follows a one-compartment bolus model with first-order output. The short half-life (5 minutes) of ADAC in plasma after intravenous injection is most likely due to rapid distribution in tissues, and it may explain a lack of side effects reported previously [19, 20]. ADAC was detected in the cochlea 5 min after administration, but the low sensitivity of the UV-Vis detection precluded the pharmacokinetic study after systemic administration. The peak cochlear level of ADAC detected following intravenous administration was consistent with pharmacological action, given the high affinity of A_1_ receptors for ADAC [5]. More sensitive method of detection, such as LC/MS, will be required to characterise ADAC concentrations in cochlear perilymph with different delivery routes.

In NIHL, postinsult time to the initiation of the treatment is an important factor which significantly affects treatment outcomes. Oxidative stress and the formation of reactive oxygen species (ROS) and reactive nitrogen species (RNS) in the cochlea is one of the major mechanisms of cochlear injury during and after noise exposure [30]. ROS levels increase postexposure, due to heavy cellular energy demands and reperfusion [30]. Production of superoxide and other free radicals reaches a maximum 7 to 10 days after exposure [31], and the production of ROS/RNS correlates with a gradual spread of hair cell loss postexposure. The peroxidation of membrane lipids, along with oxidative damage to DNA and cellular proteins, results in cell death after noise exposure [31]. Oxidative stress and other cellular events, such as inflammation and calcium overload, contribute to the development of cochlear injury after exposure to traumatic noise but also provide a window of opportunity to treat cochlear injury postexposure. We have previously shown that adenosine receptor agonists provide an effective postexposure treatment of acoustic injury and NIHL [18, 19], and the present study defines the window of opportunity for cochlear rescue. ADAC treatment provides partial rescue in the first 24 hours after noise exposure, but after that cochlear injury becomes less responsive to treatment. This is consistent with other studies demonstrating partial recovery of auditory thresholds in the first 24 hours after exposure to traumatic noise after administration of antioxidants [32–34] or antiapoptotic agents [35]. Despite the fact that free radical production appears to continue for up to 10 days after exposure, it appears that oxidative stress rapidly leads to irreversible cochlear injury and the substantial loss of critical tissues such as sensory hair cells beyond the first 24 hours, which limits the window of opportunity for pharmacological treatment of hearing loss from acute noise exposure.

**Figure 5:**
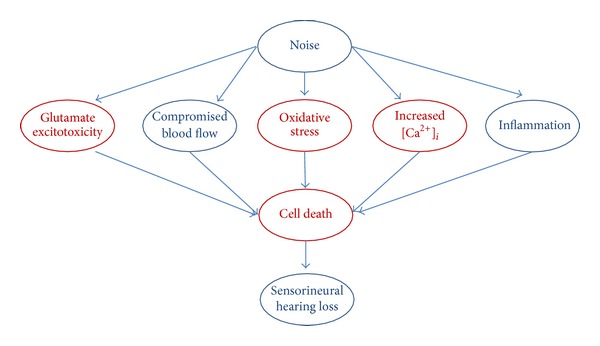
Overview of the basic mechanisms of noise-induced cochlear injury and proposed model of cochlear protection by ADAC. Putative therapeutic targets of ADAC are shown in red.

The basic cochlear protection model by ADAC has been summarised in Figure 5. Acoustic trauma can induce excessive generation of free radicals in the cochlea by overdriving the mitochondria; it can reduce cochlear blood flow, cause excitotoxic swelling of afferent nerve terminals, induce intracellular Ca^2+^ overload in sensory hair cells, and cause inflammation in cochlear tissues [24, 36]. Cellular damage results in cell death from a combination of necrosis and apoptosis, which leads to hearing loss (Figure 5). Our experimental evidence suggests that ADAC can reduce oxidative stress in the noise-exposed cochlea, leading to protection of sensory hair cells [19]. We have also demonstrated that ADAC can reduce cisplatin-induced apoptosis in cochlear tissues, particularly in sensory hair cells and strial marginal cells [25]. Other putative mechanisms of otoprotection by ADAC include inhibition of glutamate release via presynaptic A_1_ receptors and inhibition of voltage-gated Ca^2+^ channels, which can prevent activation of apoptotic and necrotic cell death pathways [14, 36]. However, further studies are required to fully understand the otoprotective mechanisms of ADAC, particularly with regard to cochlear afferent neurons.

## 5. Conclusion

Our study suggests that ADAC has a potential to be developed as a clinical otological treatment for acute hearing loss caused by exposure to traumatic noise. We show that systemic administration route is effective in mitigating cochlear injury. Previous studies suggest that the use of ADAC is not contraindicated by cardiovascular side effects at the doses used for otoprotection and neuroprotection; however, clinical trials will be necessary to confirm its safety for human use. Currently, the intratympanic administration to achieve uptake via the round window is a preferred otological drug development pathway, as it obviates possible systemic side effects (unlikely to be a factor with ADAC, but of significant regulatory body concern), and this also counters metabolism/elimination. On the other hand, intratympanic drug delivery requires expert otological intervention, whereas systemic drug administration would be more practical in a broader range of clinical situations, or circumstances where specialist surgical intervention is untenable. This is particularly relevant given that the 24-hour therapeutic window that this study indicates is therapeutically effective. Future studies are thus required to establish the optimal drug delivery method but also to establish the mechanisms of action and the optimum usage situations for ADAC. The therapeutic efficacy of ADAC for NIHL should also be evaluated in other animal models.

## Figures and Tables

**Table 1:** Chromatographic conditions

Min	%B	Flow rate (mL/min)
0	20	0.4
1	20	0.4
5	40	0.4
5.5	90	0.6
6.5	90	0.6
7	20	0.6
9.5	20	0.6
10	20	0.4
